# Investigation of the relationship between self-compassion, interpersonal problem-solving skills, and authenticity level of midwifery students

**DOI:** 10.1590/1980-220X-REEUSP-2025-0062en

**Published:** 2025-08-04

**Authors:** Esra Cevik, Sinem Ceylan

**Affiliations:** 1 Balikesir University, Faculty of Health Sciences, Department of Midwifery, Balikesir, Turkey.; 2 Ankara Medipol University, Faculty of Health Sciences, Department of Midwifery, Ankara, Turkey.

**Keywords:** Midwifery, Self-compassion, Problem Solving, Obstetrícia, Autocompaixão, Resolução de Problemas

## Abstract

**Objective:**

This study examined the relationship between midwifery students’ self-compassion and interpersonal problem-solving skills and their level of authenticity.

**Method:**

The cross-sectional study was conducted between February and September 2023. The study population consisted of 531 midwifery students. The data consisted of a descriptive characteristics form, an authenticity scale, a self-compassion scale, and an interpersonal problem-solving inventory. Univariate analyses used a t-test, ANOVA (post hoc: Tukey HSD), and linear regression.

**Results:**

The mean score on the Authenticity Scale was 46.90 ± 11.35. As the mean score of the Self-compassion Scale and the mean score of the Interpersonal Problem-Solving Inventory decreased, the Authenticity Scale score also decreased.

**Conclusion:**

The authenticity level of the participants was moderate, and the level of authenticity increased as their interpersonal problem-solving skills and self-compassion increased. It is recommended that students’ physical activity levels be increased, participation in clubs/communities should be encouraged, and studies on problem-solving skills should be provided in their education.

## RESUMO

**Objetivo:**

Este estudo examinou a relação entre a autocompaixão e as habilidades de resolução de problemas interpessoais de estudantes de obstetrícia e seu nível de autenticidade.

**Método:**

O estudo transversal foi realizado entre fevereiro e setembro de 2023. A população do estudo consistiu em 531 estudantes de obstetrícia. Os dados consistiram em um formulário de características identificadoras, uma escala de autenticidade, uma escala de autocompaixão e um inventário de resolução de problemas interpessoais. As análises univariadas utilizaram o teste T, ANOVA (post hoc: Tukey HSD) e regressão linear.

**Resultados:**

A pontuação média na Escala de Autenticidade foi de 46,90 ± 11,35. À medida que a pontuação média da Escala de Autocompaixão e a pontuação média do Inventário de Resolução de Problemas Interpessoais diminuíram, a pontuação da Escala de Autenticidade também diminuiu.

**Conclusão:**

O nível de autenticidade dos participantes foi moderado, e o nível de autenticidade aumentou à medida que suas habilidades de resolução de problemas interpessoais e autocompaixão aumentaram. Recomenda-se que os níveis de atividade física dos alunos sejam aumentados, que a participação em clubes/comunidades seja incentivada e que estudos sobre habilidades de resolução de problemas sejam fornecidos em sua educação.

## INTRODUCTION

Authenticity is being the individual’s own^(1,2,3)^, that is, the individual’s revealing his/her true self without hindrance. In other words, authenticity is the ability to harmonize one’s actions with one’s true self and is an essential factor that positively affects positive mental health^(4,5)^. Recently, the lack of authenticity due to the rapidly developing technology changing the social environment has attracted attention. Authenticity includes words such as reliable, believable, and genuine^(6)^, and authentic relationships are defined as sincere, natural, reliable, and loving communication. From this perspective, one of the essential components of the midwifery profession is authenticity.

Midwives need to establish authentic relationships with the groups they care for regarding the quality of care. Because health professionals with high authenticity skills communicate better with their patients and approach them positively^(1,6)^, it is essential for midwives, who have an important place in women-centered care, to be midwives who know themselves and present their true selves for quality health service delivery. Thus, they will better understand the needs of individuals and offer their potential for quality care. In this context, issues related to authenticity should be included in the education period of midwifery students because the rate of stress, anxiety, and depression is low in individuals with high levels of authenticity^(2)^. In a study conducted with 94 surgeons in the United States, high levels of authenticity were associated with lower burnout and depression^(4)^. A study conducted on university students from the United States and China showed that the relationships between authenticity and negative psychological health are similar across cultures^(5)^.

Midwifery is a profession that requires authenticity as well as self-compassion and compassion. Midwives need to cope with anxiety, pain, fear, and sometimes grief, as well as witnessing excitement and happiness when caring for women and their families. They must manage emotions to support women^(7)^. In this context, midwives with high authenticity, self-compassion, and interpersonal problem-solving skills are more likely to cope with these situations. Indeed, self-compassion means being kind and understanding towards oneself and enduring painful thoughts and feelings with conscious awareness^(8)^. In other words, it is an emotionally positive approach to oneself^(9)^. Those with high self-compassion are equally kind to others, while those with low self-compassion treat others more kindly than themselves^(10,11)^. Therefore, midwives with high self-compassion are more likely to show empathy. A study on midwifery students at university reported that students with high self-compassion scores experienced less compassion fatigue and burnout^(11)^. It is stated that individuals with high levels of self-compassion have more life satisfaction and higher emotional intelligence. In addition, it has been observed that self-criticism, depression, anxiety, and rumination have decreased and that they show more happiness and optimistic personality traits^(8,10)^. It has been found that individuals with self-awareness have a more robust psychological health than mindfulness, and this is associated with academic success^(8)^.

In addition to professional knowledge, midwifery students must gain critical thinking and problem-solving skills. Problem-solving skills are needed in planning, implementing, and evaluating the results of midwifery interventions^(12)^. Problem-solving skills are the efforts of the individual in the face of obstacles or situations that the individual sees as a problem^(13)^. In this context, individuals with problem-solving skills can manage care processes better and are more successful in communication skills^(14)^. Therefore, it is necessary to develop strategies to enable students to become authentic, self-compassioned individuals with interpersonal problem-solving skills to increase teaching success. In addition, determining the level of authenticity of students and revealing the relationship between problem-solving skills,self-compassion, and authenticity will provide important information about the measures to be taken to increase success in the teaching process and guide the development of strategies to facilitate learning.

Although there are studies on the level of authenticity in the literature, there are no studies conducted on midwifery students, so this study was conducted to determine the level of authenticity in midwifery students and to reveal the relationship between the level of self-compassion and problem-solving skills and the level of authenticity.

## METHOD

### Design of Study

This is a cross-sectional study.

### Sample Definition

The study interviewed midwifery students studying at a state and a foundation university between February and September 2023. The study method complies with the Strengthening the Reporting of Observational Studies in Epidemiology (STROBE) checklist. The study population consisted of 531 midwifery students studying in a state (n = 356) and a foundation university (n = 175). The sample was calculated as 355 in the Epiinfo 7.2 program by considering 50% prevalence, 95% confidence level, and 3% deviation, and 407 people were reached according to the inclusion and exclusion criteria.

### Data Collection

The study’s dependent variable is the level of authenticity, and the independent variables are sociodemographic characteristics, self-sensitivity, and interpersonal problem-solving level.

The data tools consisted of: a descriptive characteristics form, an authenticity scale, a self-compassion scale, and an interpersonal problem-solving inventory.

Descriptive Characteristics Form: The form consists of 17 questions created by the researchers based on the literature, such as age, grade, graduated high school, parent’s education level, willingly preferring the department, liking the profession, the settlement where most of the life is spent, the cohabitation status of the mother and father, the level of perception of the profession as complex, the reasons why it is complicated, long-term plans for the profession, Grade Point Average (GPA) status^(1,12,14)^.

Authenticity Scale: The scale was developed by Wood et al.^(15)^, adapted into Turkish by İlhan and Ozdemir^(16)^, and consists of 12 items in 7-point Likert type (1 = does do not define me at all; 7 = defines me completely). The scale consists of self-alienation (2, 7, 10, 12), acceptance of external influence (3, 4, 5, 6), and authentic life (1, 8, 9, 11) sub-dimensions. The lowest score on the scale is 12, and the highest is 84. Cronbach Alpha internal consistency coefficients for the sub-dimensions of the scale were 0.79–0.90 in the development study, 0.69–0.78 in the Turkish adaptation study, and 0.70–0.79 in this study.

Self-Compassion Scale: The scale was developed by Neff^(10)^ and adapted into Turkish by Akın et al.^(17)^ and consisted of 26 items in 5-point Likert type (1 = never, 2 = occasionally, 3 = about half of the time, 4 = fairly often, 5 = always) and six dimensions: self-compassion, self-judgment, awareness of sharing, isolation, consciousness, and over-identification. Scale means a total score between 1–2.5 indicates low self-compassion, 2.5–3.5 indicates moderate self-compassion, and 3.5–5 indicates high self-compassion. The Cronbach Alpha internal consistency coefficients for the subscales of the scale were between 0.75–0.81 in the development study, 0.72–0.80 in the Turkish adaptation study, and 0.70–0.76 in this study.

Interpersonal Problem-Solving Inventory: The scale was developed by Çam and Tumkaya^(13)^ on a 5-point Likert scale (1 = not at all appropriate, 2 = somewhat appropriate, 3 = appropriate, 4 = mostly appropriate, 5 = completely appropriate). It consists of 50 items and 5 sub-dimensions: negative approach to the problem, constructive problem solving, self-distrust, not taking responsibility, and insistent- persistent approach. The lowest score that can be obtained from the scale is 50, and the highest score is 250. The high score obtained for each subscale indicates that the trait related to interpersonal problem-solving is high. Cronbach’s alpha internal consistency coefficient was 0.91 in the development study of the scale and 0.931 in our study.

### Data Analysis and Treatment

Data were collected with an online questionnaire created through Google Forms. Midwifery students aged 18 years and over who agreed to participate in the study were included. The data obtained in the study were evaluated using the IBM SPSS Statistics for Windows, Version 24.0. The descriptive analysis used numbers, percentages, mean, and standard deviation. The normality assumption of continuous data was evaluated according to the Shapiro-Wilk test, and the kurtosis skewness coefficient was between +1.5 and −1.5. The t-test was used to compare two group averages, the One-Way ANOVA (post hoc: Tukey HSD) test was used to compare three or more group averages, and the Pearson correlation test was used to evaluate the relationship between continuous variables. Variables found significant in univariate analyses were evaluated by linear regression analysis. The results have been evaluated at a 95% confidence interval and p < 0.05 significance level.

### Ethical Aspects

In this study, the rules of the Declaration of Helsinki were followed, and permission was obtained from the ethics committee of the relevant university (approval date: 28 June 2022, approval number: 2022/287), the institutions where the research was conducted and the scale owners. An online questionnaire was created, and the data collection form was sent to the students who agreed to participate in the study. “Informed Voluntary Consent Form” was included in the online survey, and those who gave consent could access the questions.

## RESULTS

The mean age of the research group (n = 407) was 20.64 ± 1.61; 51.8% were 21 or older, 67.3% were State university students, and 32.4% were first-year students. The mean GPA of the participants was 2.91 ± 0.46; 51.8% had a GPA between 0–2.99, 66.1% had 1–2 siblings, 74% had a middle-income perception, 33.2% spent most of their lives in metropolitan areas, 92.1% had parents living together, 49.1% had a mother who graduated from primary school, and 33.7% had a father who graduated from primary school. 12.5% of the participants smoke cigarettes and 10.8% drink alcohol. In addition, 21.4% of the participants stated that they had experienced some form of violence in the last 6 months, 63.6% spent 0-3 hours a day on social media, 43.7% exercised 1-2 hours a week, 28.5% were members of a club/community, and 79.4% chose the midwifery department willingly.

In the research group, the mean score of the Authenticity Scale was higher in State University students (t = −2.456, p = 0.015), in those with a GPA of 3 and above (t = 2.610, p = 0.009), in those whose parents lived together (t = 4.341, p = 0.000), in those whose mother had high school education or above (t = 5.779, p = 0.000), in those whose father had high school education or above (t = 2. 737, p = 0.006), those who have not been exposed to some form of violence in the last 6 years (t = 10.260, p = 0.000), those who spend 4 hours or more on social media (t = −3.340, p = 0.001), those who exercise (F = 67.181, p = 0.000), those who are members of a club/community (t = 5.545, p = 0.000), and those who choose the midwifery program willingly (t = 9.049, p = 0.000). There is no statistically significant difference (p > 0.05) according to age, class, number of siblings, income perception, place of residence, family structure, smoking, and alcohol use variables (Table 1).

**Table 1:** Sociodemographic characteristics of the research group and mean scores of the authenticity scale according to sociodemographic characteristics – Balikesir, Ankara, Turkey, 2023.

Variables	n	%	X ± SD	Test value	p
**Age X ± SD** 20.64 ± 1.61					
18–20 years old	196	48.2	46.81 ± 10.96	t = 0.150	0.881
21 years and older	211	51.8	46.98 ± 11.73		
**University**					
Foundation	133	32.7	45.05 ± 9.89	t = −2.456	**0.015**
State	274	67.3	47.80 ± 11.91		
**Grade**					
1	132	32.4	46.82 ± 10.86	F = 0.370	0.774
2	116	28.5	46.75 ± 11.56		
3	99	24.3	46.36 ± 11.29		
4	60	14.7	48.26 ± 12.24		
**GPA X ± SD** 2.91 ± 0.46					
0–2.99	211	51.8	45.49 ± 10.64	t = 2.610	**0.009**
3 and above	196	48.2	48.42 ± 11.91		
**Number of siblings**					
0–2	285	70.0	47.02 ± 11.67	t = 0.317	0.751
3 and above	122	30.0	46.63 ± 10.60		
**Income perception**					
High	31	7.6	45.06 ± 9.04	F = 0.440	0.645
Middle	301	74.0	47.06 ± 11.32		
Low	75	18.4	47.02 ± 12.34		
**Where the majority of like takes place**					
Metropolitan	135	33.2	47.42 ± 11.84	F = 0.313	0.816
City	98	24.1	46.06 ± 10.39		
District	113	27.8	46.76 ± 10.56		
Village	61	15.0	47.37 ± 13.19		
**Family structure**					
Parents together	375	92.1	47.60 ± 11.00	t = 4.341	**0.000**
Divorced/Deceased	32	7.9	38.71 ± 12.30		
**Mother’s education level**					
Primary school and below	234	57.4	44.08 ± 9.10	t = 5.779	**0.000**
High school and above	173	42.6	50.71 ± 12.90		
**Father’s education level**					
Primary school and below	153	37.6	45.06 ± 9.25	t = 2.737	**0.006**
High school and above	254	62.4	48.01 ± 12.33		
**Cigarette smoking**					
Yes	51	12.5	49.05 ± 11.73	t = −1.409	0.164
No	356	87.5	46.59 ± 11.28		
**Alcohol use**					
Yes	44	10.8	48.25 ± 10.02	t = −0.832	0.406
No	363	89.2	46.74 ± 11.50		
**Experiencing some form of violence (in the last 6 months)**					
Yes	87	21.4	38.60 ± 7.62	t = 10.260	**0.000**
No	320	78.6	49.15 ± 11.15		
**Time spent on social media (per day)**					
0–3 hours	259	63.6	45.49 ± 11.18	t = −3.340	**0.001**
4 hours or more	148	36.4	49.36 ± 11.26		
**Exercise (per week)**					
Never^a^	90	22.1	37.95 ± 10.26	F = 67.181	**0.001** a<b<c
1–2 hours^b^	178	43.7	46.38 ± 8.10		
3 hours and over^c^	139	34.2	53.36 ± 11.50		
**Club/community membership**					
Yes	116	28.5	52.40 ± 13.70	t = 5.545	**0.000**
No	291	71.5	44.71 ± 9.43		
**Choosing the midwifery department willingly**					
Yes	323	79.4	49.27 ± 10.30	t = 9.049	**0.000**
No	84	20.6	37.78 ± 10.58		

**X:** Mean, **SD:** Standard deviation, **t**: Student’s t-test, **F**: Oneway ANOVA (post hoc: Tukey HSD), **GPA:** Grade Point Average.

The mean age of the participants was 20.64 ± 1.61. The mean GPA was 2.91 ± 0.47, the mean number of siblings was 2.26 ± 1.85, the mean number of cigarettes smoked per day was 8.47 ± 4.77, the mean number of glasses of alcohol consumed per week was 9.93 ± 9.49, the mean score of the Authenticity Scale was 46.90 ± 11.35, the mean score of the Self Alienation sub-dimension was 14. 11 ± 4.41, Acceptance of External Influence subscale mean score 19.46 ± 4.22, Authentic Life subscale mean score 13.32 ± 5.63, Self-Compassion Scale mean score 2.79 ± 0.48, Self-affection subscale mean score 2.96 ± 0.81, Self-judgment subscale mean score 2.43 ± 0. 92, Consciousness of Sharing subscale mean score 2.92 ± 0.77, Isolation subscale mean score 2.68 ± 0.95, Consciousness subscale mean score 3.03 ± 0.83, Overidentification subscale mean score 2.77 ± 0.90, Interpersonal Problem-Solving Inventory mean score 125.33 ± 21. 27, the mean score of the Negative Approach to Problem dimension was 38.47 ± 11.05, the mean score of the Constructive problem-solving dimension was 45.38 ± 8.45, the mean score of the Insecurity dimension was 13.34 ± 4.84, the mean score of the Taking Responsibility dimension was 10.66 ± 3.32. The mean score of the Insistent-Persistent Approach sub-dimension was 17.47 ± 3.37 (Table 2).

**Table 2:** Distribution of continuous variables in the study group – Balikesir, Ankara, Turkey, 2023.

Variables	X ± SD	Min–Max
Age	20.64 ± 1.61	18–32
GPA	2.91 ± 0.47	1.00–3.90
Number of siblings	2.26 ± 1.85	0–13
Number of cigarettes (per day)	8.47 ± 4.77	1–20
Alcohol quantity (per week)	9.93 ± 9.49	1–52
Authenticity Scale	46.90 ± 11.35	12–81
Self Alienation	14.11 ± 4.41	4–28
Acceptance of External Influence	19.46 ± 4.22	4–28
Authentic Life	13.32 ± 5.63	4–28
Self-Compassion Scale	2.79 ± 0.48	1–4.54
Self-Affection	2.96 ± 0.81	1–5
Self-Judgment	2.43 ± 0.92	1–5
Awareness of Sharing	2.92 ± 0.77	1–5
Isolation	2.68 ± 0.95	1–5
Consciousness	3.03 ± 0.83	1–5
Over-identification	2.77 ± 0.90	1–5
Interpersonal Problem-Solving Inventory	125.33 ± 21.27	50–200
Negative Approach to the Problem	38.47 ± 11.05	16–64
Constructive Problem Solving	45.38 ± 8.45	16–64
Self Insecurity	13.34 ± 4.84	7–28
Lack of Responsibility	10.66 ± 3.32	5–20
Insistent-Persistent Approach	17.47 ± 3.37	50–200

X: Mean, SD: Standard deviation, GPA: Grade Point Average.

When the relationship between the continuous variables and the Authenticity Scale in the research group was analyzed, it was found that there was no statistically significant difference between the variables of GPA (r = 0.111, p = 0.026), Self-Compassion score (r = 0.371, p = 0. 000), Self-judgment score (r = 0.570, p = 0.000), Isolation score (r = 0.5022, p = 0.000), Overidentification score (r = 0.492, p = 0.000), Interpersonal Problem-Solving score (r = 0.397, p = 0.000), Negative Approach to Problems score (r = 0.491, p = 0.000), Insecurity score (r = 0.296, p = 0.000), Lack of Responsibility score (r = 0.197, p = 0.000), Insistent-Persistent Approach score increases, Conscientiousness score (r = −0.179, p = 0.000) and Self-Compassion score decreases (r = −0.207, p = 0.000), Authenticity Scale score increases statistically significantly. There is no significant difference according to other variables and dimensions (p > 0.05) (Table 3).

**Table 3:** The relationship between continuous variables and authenticity level – Balikesir, Ankara, Turkey, 2023.

Variables	r	p
Age	−0.003	0.948
GPA	0.111	0.026
Number of siblings	−0.056	0.260
Number of cigarettes	−0.179	0.209
Alcohol quantity	−0.208	0.175
Self-Compassion Scale	0.371	0.000
Self-Affection	−0.207	0.000
Self-Judgment	0.570	0.000
Awareness of Sharing	−0.077	0.121
Isolation	0.522	0.000
Consciousness	−0.179	0.000
Over-identification	0.492	0.000
Interpersonal Problem-Solving Inventory	0.397	0.000
Negative Approach to the Problem	0.491	0.000
Constructive Problem Solving	0.056	0.261
Self Insecurity	0.296	0.000
Lack of Responsibility	0.197	0.000
Insistent-Persistent Approach	0.132	0.008

X: Mean, SD: Standard deviation, r: Pearson’s correlation coefficient, GPA: Grade Point Average.

The variables that were statistically significant because of univariate analyses and the relationship between the significant independent variables in the literature and the dependent variable were evaluated by linear regression analysis using the Enter method. The variables included in the model explain 52% of the change in the Authenticity Scale (R2 = 0.538, Adjusted R2 = 0.524, F = 38.206 p = 0.000).

As a result of the linear regression analysis, the score of the Authenticity Scale was found to be significantly lower in students studying at a foundation university (β = 2.671; 95% CI 0.96; 4.37), in those whose parents were separated/dead (β = −5.083; 95% CI −7.99; −2.17), in those whose mothers had primary education or less (β = −4.478; 95% CI −6.28; −2.67), in those who experienced some form of violence in the last 6 months (β = −5. 272; 95% CI −7.23; −3.30), those who spend less time on social media (β = 1.006; 95% CI 0.46; 1.54), those whose exercise duration decreases (β = −2.648; 95% CI −3.43; −1. 86), those who were not members of a club/community (β = −3.236; 95% CI −5.00; −1.46), and those who did not choose the midwifery program willingly (β = −5.383; 95% CI −7.47; −3.29). In addition, as the Self-Compassion score decreases (β = 0.102; 95% CI 0.02; 0.17) and the Interpersonal Problem-Solving score decreases (β = 0.081; 95% CI 0.03–0.12), the Authenticity score of the participants also decreases (Table 4).

**Table 4:** Evaluation of independent variables and authenticity score according to linear regression analysis – Balikesir, Ankara, Turkey, 2023.

Variables	ß	S.E.	Std. ß	p	%95 C.I.	
					Lower	Upper
University	2.671	0.867	0.110	0.002	0.96	4.37
GPA	0.957	0.863	0.039	0.268	−0.73	2.65
Family structure	−5.083	1.482	−0.121	0.001	−7.99	−2.17
Mother’s education level	−4.478	0.918	−0.195	0.000	−6.28	−2.67
Father’s education level	1.505	0.913	0.064	0.100	−0.29	3.29
Experiencing some form of violence in the last 6 months	−5.272	1.000	−0.191	0.000	−7.23	−3.30
Time spent on social media (per day)	1.006	0.276	0.127	0.000	0.46	1.54
Exercise (per week)	−2.648	0.399	−0.258	0.000	−3.43	−1.86
Club/community membership	−3.236	0.900	−0.129	0.000	−5.00	−1.46
Choosing the midwifery department willingly	−5.383	1.063	−0.192	0.000	−7.47	−3.29
Self-Compassion Scale	0.102	0.039	0.114	0.010	0.02	0.17
Interpersonal Problem-Solving Inventory	0.081	0.024	0.152	0.001	0.03	0.12

ß: Regression coefficient, S.E.: Standard error, p: significance value, CI: Confidence interval, GPA: Grade Point Average. Adjusted R2 = 0.524, F = 38.206, p = 0.000.

## DISCUSSION

Authentic individuals are creative, open to innovation, and trust their experiences and inwardness^(18)^. The ability of midwives to be authentic individuals significantly affects the quality of care, interprofessional cooperation, organizational commitment, and job satisfaction^(6,19,20)^. In this study, which was conducted to determine the relationship between self-compassion, interpersonal problem-solving skills, and authenticity levels in midwifery students, it was determined that there was a significant positive relationship between self-compassion and problem-solving skills and authenticity levels of midwifery students. In our study, the level of authenticity of midwifery students was found to be medium, concurring with the literature. The fact that authenticity is not high may be due to the limited opportunities for practice due to the increasing number of students, the inability of midwifery students to spend limited time with women in the field of practice in Turkey, and the status of midwifery. In the study conducted by Bloxsome and Bayes, the importance of staying with women is emphasized to feel innate identity, identity arising from the environment, and authenticity^(21)^.

In addition, authenticity scores increase with GPA. This result suggests that students’ success in midwifery education also improves their authenticity. There is no study in the literature examining the authenticity level of midwifery students. On the other hand, studies examine university students’ authenticity levels. These studies were mainly conducted on students in the field of social sciences. These studies’ results are similar to ours, and the authenticity score of university students was determined to be at a medium level^(1,2,3,22)^. When the studies conducted with different methods in the literature are examined, it is seen that authenticity increases as the midwifery students’ relationships with health service providers increase^(23)^ and as the application opportunities such as simulation increase^(24)^. In this respect, attempts should be made to increase the authenticity of midwifery students.

Our study found that age, grade, number of siblings, income perception, place of residence, family structure, smoking status, and alcohol use did not affect authenticity. Although there are no studies on midwifery students in the literature, it is seen that the age of nursing students and nurses affects the level of authenticity^(1,25)^. Midwifery education requires a high level of professional knowledge and skills due to the nature of the profession; therefore, it is one of the professions where anxiety about being in a different educational environment and emotional burnout are frequently experienced^(26)^. Measuring students’ self-compassion during their education, determining the factors affecting it, and increasing it will increase the professional development of midwifery students. In the literature, it is reported that the higher the level of authenticity, the higher the self-sensitivity of individuals^(18,27)^_._ In our study, as the level of self-compassion increases, the level of authenticity also increases. From this point of view, it can be said that being a good friend with oneself increases the ability to act in accordance with one’s feelings and thoughts as independently as possible from others by revealing the true self. In a study conducted with nursing students in the literature similar to this study, it was found that authenticity increased as self-compassion increased^(1)^. A similar result was found in our study, and as the self-compassion of midwifery students increased, so did their level of authenticity. As a result of a study conducted on university students, it was determined that authenticity sub-dimensions explained 28% of the self-compassion level^(18)^. Adding subjects that improve self-compassion to the education programs of midwifery students can prepare them to manage stress factors, promote self-care, and improve compassionate care provided to others.

Providing individualized midwifery care requires critical and analytical thinking skills, creativity, and the ability to produce practical solutions to the problems they face. In this context, midwifery students need learning experiences in which a problem-solving approach is adopted to develop their knowledge, skills, and attitudes. In our study, midwifery students were found to have moderate problem-solving skills. When the problem-solving skills of midwifery students were examined in the literature, it was determined that their problem-solving skills were at a medium level^(12,14,28)^. However, no literature study examines the effect of problem-solving skills on authenticity. In our study, it was found that the level of authenticity increased as problem-solving skills increased. This situation is interpreted as students planning creative and innovative midwifery care as they improve their problem-solving skills. In this context, it is recommended that multiple teaching methods such as simulation-based learning, applied learning, reflective use, and project-based learning, which increase problem-solving skills, be used to increase midwifery students’ problem-solving, self-direction, and authenticity and be integrated into university education.

To evaluate this study correctly, some limitations must be considered. First, a limitation of study is that the findings are based on self-reported data (self-completed questionnaires). Secondly, this study only included a limited number of midwifery students from two different universities, so the authors acknowledge that the sample is unlikely to be representative of the population. In future research, it is recommended that similar research be conducted with larger and more diverse samples.

## CONCLUSION

The findings revealed that participants had moderate levels of authenticity, while their self-compassion and problem-solving skills were low. Increases in self-compassion and interpersonal problem-solving were found to positively influence authenticity. The factors affecting authenticity were: university where the participant was trained; family structure; maternal education level; exposure to some form of violence in the last 6 months; time spent on social media; club membership, and choosing the profession willingly. As interpersonal problem-solving skills and self-compassion increase, authenticity also increases.

It is recommended to ensure family integrity, increase parental education, increase students’ physical activity levels, encourage participation in clubs/communities, and increase problem-solving and sensitivities.

It is recommended that educational programs integrate multiple teaching methods—such as simulation-based, applied, reflective, and project-based learning—to enhance students’ authenticity, self-compassion, and problem-solving skills. Including interventions targeting self-compassion and problem-solving in individual/group counseling and psychoeducational practices may also be effective. Future experimental studies are recommended to evaluate the impact of such programs on authenticity, and research should be extended to different age groups and educational levels.

## Data Availability

Data related to this study are available from the corresponding author upon justified request.
