# Supplementary material for: Investigation of the relationship between self-compassion, interpersonal problem-solving skills, and authenticity level of midwifery students
Source: Rev Esc Enferm USP. 2025 Aug 4;59:e20250062. doi: 10.1590/1980-220X-REEUSP-2025-0062en (PMC12323389; doi:10.1590/1980-220X-REEUSP-2025-0062en)
Supplement: Translation

# Translation: Investigación de la relación entre la autocompasión, las habilidades de resolución de problemas interpersonales y el nivel de autenticidad de estudiantes de partería

**DOI:** 10.1590/1980-220X-REEUSP-2025-0062es

**Authors:** Esra Cevik, Sinem Ceylan

**Affiliations:** 1 Balikesir University, Faculty of Health Sciences, Department of Midwifery, Balikesir, Turkey.; 2 Ankara Medipol University, Faculty of Health Sciences, Department of Midwifery, Ankara, Turkey.

**Keywords:** Partería, Autocompasión, Solución de Problemas

## Abstract

**Objetivo:**

Este estudio examinó la relación entre la autocompasión y las habilidades de resolución de problemas interpersonales de los estudiantes de partería y su nivel de autenticidad.

**Método:**

El estudio transversal se llevó a cabo entre febrero y septiembre de 2023. La población de estudio consistió en 531 estudiantes de partería. Los datos consistieron en un formulario de características de identificación, una escala de autenticidad, una escala de autocompasión y un inventario de resolución de problemas interpersonales. Los análisis univariados utilizaron una prueba T, ANOVA (post hoc: Tukey HSD) y regresión lineal.

**Resultados:**

La puntuación media de la Escala de Autenticidad fue de 46,90 ± 11,35. A medida que disminuía la puntuación media de la Escala de Autocompasión y la puntuación media del Inventario de Resolución de Problemas Interpersonales, también disminuía la puntuación de la Escala de Autenticidad.

**Conclusiones:**

El nivel de autenticidad de los participantes fue moderado, y el nivel de autenticidad aumentó a medida que aumentaron sus habilidades de resolución de problemas interpersonales y su autocompasión. Se recomienda aumentar los niveles de actividad física de los estudiantes, fomentar la participación en clubes/comunidades y proporcionar estudios sobre habilidades de resolución de problemas en su educación.

## INTRODUCCIÓN

La autenticidad es ser el propio individuo^(1,2,3)^, es decir, que el individuo revele su verdadero yo sin impedimentos. En otras palabras, la autenticidad es la capacidad de armonizar las acciones de uno con su verdadero yo y es un factor esencial que afecta positivamente a la salud mental positiva^(4,5)^. Recientemente, la falta de autenticidad debida al rápido desarrollo de la tecnología que cambia el entorno social ha llamado la atención. La autenticidad incluye palabras como fiable, creíble y genuino^(6)^, y las relaciones auténticas se definen como una comunicación sincera, natural, fiable y cariñosa. Desde esta perspectiva, uno de los componentes esenciales de la profesión de partería es la autenticidad.

Las parteras necesitan establecer relaciones auténticas con los grupos a los que atienden en lo que respecta a la calidad de los cuidados. Dado que los profesionales sanitarios con un alto grado de autenticidad se comunican mejor con sus pacientes y se acercan a ellos de forma positiva^(1,6)^, es esencial que las parteras, que ocupan un lugar importante en la atención centrada en la mujer, sean matronas que se conozcan a sí mismas y presenten su verdadero yo para una prestación de servicios de salud de calidad. De este modo, comprenderán mejor las necesidades de las personas y ofrecerán su potencial para una atención de calidad. En este contexto, los temas relacionados con la autenticidad deberían incluirse en el periodo de formación de los estudiantes de partería, ya que la tasa de estrés, ansiedad y depresión es baja en individuos con altos niveles de autenticidad^(2)^. En un estudio realizado con 94 cirujanos en Estados Unidos, los altos niveles de autenticidad se asociaron con un menor agotamiento y depresión^(4)^. Un estudio realizado con estudiantes universitarios de Estados Unidos y China demostró que las relaciones entre la autenticidad y la salud psicológica negativa son similares en todas las culturas^(5)^.

La partería es una profesión que requiere autenticidad, así como autocompasión y compasión. Las parteras tienen que hacer frente a la ansiedad, el dolor, el miedo y, a veces, la pena, así como ser testigos de la emoción y la felicidad cuando atienden a las mujeres y a sus familias. Deben gestionar las emociones para apoyar a las mujeres^(7)^. En este contexto, las parteras con un alto grado de autenticidad, autocompasión y capacidad para resolver problemas interpersonales tienen más probabilidades de hacer frente a estas situaciones. De hecho, la autocompasión significa ser amable y comprensivo con uno mismo y soportar los pensamientos y sentimientos dolorosos con conciencia^(8)^. En otras palabras, es un enfoque emocionalmente positivo hacia uno mismo^(9)^. Las personas con alta autocompasión son igual de amables con los demás, mientras que las que tienen baja autocompasión tratan a los demás con más amabilidad que a sí mismas^(10,11)^. Por lo tanto, las parteras con alta autocompasión son más propensas a mostrar empatía. Un estudio sobre estudiantes universitarias de partería reveló que las estudiantes con altos niveles de autocompasión experimentaban menos fatiga por compasión y agotamiento^(11)^. Se afirma que los individuos con altos niveles de autocompasión tienen más satisfacción vital y mayor inteligencia emocional. Además, se ha observado que la autocrítica, la depresión, la ansiedad y la rumiación han disminuido y que muestran más felicidad y rasgos de personalidad optimistas^(8,10)^. Se ha comprobado que los individuos con autoconciencia tienen una salud psicológica más sólida que con mindfulness, y esto se asocia con el éxito académico^(8)^.

Además de los conocimientos profesionales, los estudiantes de partería deben adquirir habilidades de pensamiento crítico y de resolución de problemas. Las habilidades de resolución de problemas son necesarias para planificar, aplicar y evaluar los resultados de las intervenciones de partería^(12)^. Las habilidades de resolución de problemas son los esfuerzos del individuo ante obstáculos o situaciones que el individuo ve como un problema^(13)^. En este contexto, los individuos con habilidades de resolución de problemas pueden gestionar mejor los procesos asistenciales y tienen más éxito en las habilidades de comunicación^(14)^. Por lo tanto, es necesario desarrollar estrategias que permitan a los estudiantes convertirse en individuos auténticos, autocompasivos y con habilidades de resolución de problemas interpersonales para aumentar el éxito de la enseñanza. Además, determinar el nivel de autenticidad de los estudiantes y revelar la relación entre las habilidades de resolución de problemas, la autocompasión y la autenticidad proporcionará información importante sobre las medidas que deben tomarse para aumentar el éxito en el proceso de enseñanza y orientar el desarrollo de estrategias que faciliten el aprendizaje.

Aunque existen estudios sobre el nivel de autenticidad en la literatura, no hay estudios realizados en estudiantes de partería, por lo que este estudio se llevó a cabo para determinar el nivel de autenticidad en estudiantes de partería y revelar la relación entre el nivel de autocompasión y habilidades de resolución de problemas y el nivel de autenticidad.

## MÉTODO

### Diseño del Estudio

Se trata de un estudio transversal.

### Definición de la Muestra

El estudio entrevistó a estudiantes de partería que cursaban estudios en una universidad pública y en una universidad de fundación entre febrero y septiembre de 2023. El método del estudio se ajusta a la lista de verificación Strengthening the Reporting of Observational Studies in Epidemiology (STROBE). La población del estudio consistió en 531 estudiantes de partería que estudiaban en una universidad estatal (n = 356) y en una universidad de fundación (n = 175). La muestra se calculó como 355 en el programa Epiinfo 7,2 considerando una prevalencia del 50%, un nivel de confianza del 95% y una desviación del 3%, y se llegó a 407 personas de acuerdo con los criterios de inclusión y exclusión.

### Recolección de Datos

La variable dependiente del estudio es el nivel de autenticidad, y las variables independientes son las características sociodemográficas, la autosensibilidad y el nivel de resolución de problemas interpersonales.

Las herramientas de datos consistieron en: un formulario de características identificativas, una escala de autenticidad, una escala de autocompasión y un inventario de resolución de problemas interpersonales.

Formulario de características descriptivas: El formulario consta de 17 preguntas creadas por los investigadores basándose en la bibliografía, como la edad, el grado, el bachillerato terminado, el nivel educativo de los padres, la preferencia voluntaria por el departamento, el gusto por la profesión, el asentamiento donde se pasa la mayor parte de la vida, el estado de convivencia de la madre y el padre, el nivel de percepción de la profesión como compleja, las razones por las que es complicada, los planes a largo plazo para la profesión, el estado del promedio de calificaciones (GPA)^(1,12,14)^.

Escala de autenticidad: La escala fue desarrollada por Wood et al.^(15)^, adaptada al turco por İlhan y Ozdemir^(16)^, y consta de 12 ítems tipo Likert de 7 puntos (1 = no me define en absoluto; 7 = me define completamente). La escala consta de subdimensiones de autoalienación (2, 7, 10, 12), aceptación de la influencia externa (3, 4, 5, 6) y vida auténtica (1, 8, 9, 11). La puntuación más baja de la escala es 12, y la más alta 84. Los coeficientes de consistencia interna Alfa de Cronbach para las subdimensiones de la escala fueron 0,79–0,90 en el estudio de desarrollo, 0,69–0,78 en el estudio de adaptación turco, y 0,70–0,79 en este estudio.

Escala de autocompasión: La escala fue desarrollada por Neff^(10)^ y adaptada al turco por Akın et al.^(17)^ y constaba de 26 ítems tipo Likert de 5 puntos (1 = nunca, 2 = ocasionalmente, 3 = aproximadamente la mitad de las veces, 4 = bastante a menudo, 5 = siempre) y seis dimensiones: autocompasión, autojuicio, conciencia de compartir, aislamiento, conciencia y sobreidentificación. Según la escala, una puntuación total entre 1–2,5 indica baja autocompasión, 2,5–3,5 indica moderada autocompasión y 3,5–5 indica alta autocompasión. Los coeficientes de consistencia interna Alfa de Cronbach para las subescalas de la escala se situaron entre 0,75–0,81 en el estudio de desarrollo, 0,72–0,80 en el estudio de adaptación turco, y 0,70–0,76 en este estudio.

Inventario de resolución de problemas interpersonales: La escala fue desarrollada por Çam y Tumkaya^(13)^ en una escala Likert de 5 puntos (1 = nada apropiado, 2 = algo apropiado, 3 = apropiado, 4 = mayormente apropiado, 5 = completamente apropiado). Consta de 50 ítems y 5 subdimensiones: enfoque negativo del problema, resolución constructiva de problemas, desconfianza en uno mismo, no asumir la responsabilidad y enfoque insistente-persistente. La puntuación más baja que puede obtenerse de la escala es 50, y la más alta 250. La alta puntuación obtenida en cada subescala indica que el rasgo relacionado con la resolución de problemas interpersonales es alto. El coeficiente de consistencia interna alfa de Cronbach fue de 0,91 en el estudio de desarrollo de la escala y de 0,931 en nuestro estudio.

### Análisis y Tratamiento de los Datos

Los datos se recolectaron con un cuestionario online creado a través de Google Forms. Se incluyeron estudiantes de Partería mayores de 18 años que aceptaron participar en el estudio. Los datos obtenidos en el estudio se evaluaron utilizando el programa IBM SPSS Statistics para Windows, versión 24,0. En el análisis descriptivo se utilizaron números, porcentajes, media y desviación estándar. El supuesto de normalidad de los datos continuos se evaluó según la prueba de Shapiro-Wilk, y el coeficiente de asimetría de la curtosis se situó entre +1,5 y –1,5. Se utilizó la prueba T para comparar las medias de dos grupos, la prueba ANOVA unidireccional (post hoc: Tukey HSD) para comparar las medias de tres o más grupos, y la prueba de correlación de Pearson para evaluar la relación entre variables continuas. Las variables que resultaron significativas en los análisis univariantes se evaluaron mediante un análisis de regresión lineal. Los resultados se han evaluado con un intervalo de confianza del 95% y un nivel de significación p < 0,05.

### Aspectos Éticos

En este estudio se siguieron las normas de la Declaración de Helsinki y se obtuvo el permiso del comité de ética de la universidad correspondiente (fecha de aprobación: 28 de junio de 2022, número de aprobación: 2022/287), de las instituciones donde se llevó a cabo la investigación y de los propietarios de las escalas. Se creó un cuestionario en línea y se envió el formulario de recogida de datos a los estudiantes que aceptaron participar en el estudio. En la encuesta en línea se incluyó el «Formulario de consentimiento voluntario informado», y quienes dieron su consentimiento pudieron acceder a las preguntas.

## RESULTADOS

La edad media del grupo de investigación (n = 407) fue de 20,64 ± 1,61; el 51,8% tenía 21 años o más, el 67,3% eran estudiantes de la universidad estatal y el 32,4% eran estudiantes de primer curso. La nota media de los participantes era de 2,91 ± 0,46; el 51,8% tenía una nota entre 0–2,99, el 66,1% tenía 1–2 hermanos, el 74% tenía una percepción de ingresos media, el 33,2% pasaba la mayor parte de su vida en áreas metropolitanas, el 92,1% tenía padres que vivían juntos, el 49,1% tenía una madre graduada en primaria y el 33,7% tenía un padre graduado en primaria. El 12,5% de los participantes fuma cigarrillos y el 10,8% bebe alcohol. Además, el 21,4% de las participantes declara haber sufrido algún tipo de violencia en los últimos 6 meses, el 63,6% pasa entre 0 y 3 horas al día en las redes sociales, el 43,7% hace ejercicio entre 1 y 2 horas a la semana, el 28,5% es miembro de un club/comunidad y el 79,4% elige voluntariamente el departamento de partería.

En el grupo de investigación, la puntuación media de la Escala de Autenticidad fue mayor en los estudiantes de la Universidad Estatal (t = −2,456, p = 0,015), en los que tenían un GPA de 3 y superior (t = 2,610, p = 0,009), en aquellos cuyos padres vivían juntos (t = 4,341, p = 0,000), en aquellos cuya madre tenía educación secundaria o superior (t = 5,779, p = 0,000), en aquellos cuyo padre tenía educación secundaria o superior (t = 2. 737, p = 0,006), los que no han estado expuestos a algún tipo de violencia en los últimos 6 años (t = 10,260, p = 0,000), los que pasan 4 horas o más en las redes sociales (t = −3,340, p = 0. 001), las que hacen ejercicio (F = 67,181, p = 0,000), las que son miembros de un club/comunidad (t = 5,545, p = 0,000), y las que eligen el programa de partería voluntariamente (t = 9,049, p = 0,000). No hay diferencias estadísticamente significativas (p > 0,05) según las variables edad, clase, número de hermanos, percepción de ingresos, lugar de residencia, estructura familiar, tabaquismo y consumo de alcohol (Tabla 1).

**Tabla 1:** Características sociodemográficas del grupo de investigación y puntuaciones medias de la escala de autenticidad según las características sociodemográficas – Balikesir, Ankara, Turquía, 2023.

Variables	n	%	X ± DE	Valor de prueba	p
**Edad X ± DE** 20,64 ± 1,61					
18–20 años	196	48,2	46,81 ± 10,96	t = 0,150	0,881
Mayores de 21 años	211	51,8	46,98 ± 11,73		
**Universidad**					
Fundación	133	32,7	45,05 ± 9,89	t = –2,456	**0,015**
Estado	274	67,3	47,80 ± 11,91		
**Grado**					
1	132	32,4	46,82 ± 10,86	F = 0,370	0,774
2	116	28,5	46,75 ± 11,56		
3	99	24,3	46,36 ± 11,29		
4	60	14,7	48,26 ± 12,24		
**GPA X ± SD** 2,91± 0,46					
0–2,99	211	51,8	45,49 ± 10,64	t = 2,610	**0,009**
3 y superiores	196	48,2	48,42 ± 11,91		
**Número de hermanos**					
0–2	285	70,0	47,02 ± 11,67	t = 0,317	0,751
3 y superiores	122	30,0	46,63 ± 10,60		
**Percepción de ingresos**					
Alta	31	7,6	45,06 ± 9,04	F = 0,440	0,645
Medio	301	74,0	47,06± 11,32		
Bajo	75	18,4	47,02± 12,34		
**Dónde tiene lugar la mayoría de la vida**					
Metropolitano	135	33,2	47,42 ± 11,84	F = 0,313	0,816
Ciudad	98	24,1	46,06 ± 10,39		
Distrito	113	27,8	46,76 ± 10,56		
Pueblo	61	15,0	47,37 ± 13,19		
**Estructura familiar**					
Padres juntos	375	92,1	47,60 ± 11,00	t = 4,341	**0,000**
Divorciado/fallecido	32	7,9	38,71 ± 12,30		
**Nivel de estudios de la madre**					
Enseñanza primaria e inferior	234	57,4	44,08 ± 9,10	t = 5,779	**0,000**
Bachillerato y superior	173	42,6	50,71 ± 12,90		
**Nivel de estudios del padre**					
Enseñanza primaria e inferior	153	37,6	45,06 ± 9,25	t = 2,737	**0,006**
Bachillerato y superior	254	62,4	48,01 ± 12,33		
**Fumar cigarrillos**					
Sí	51	12,5	49,05 ± 11,73	t = –1,409	0,164
No	356	87,5	46,59 ± 11,28		
**Consumo de alcohol**					
Sí	44	10,8	48,25 ± 10,02	t = –0,832	0,406
No	363	89,2	46,74 ± 11,50		
**Ha sufrido algún tipo de violencia (en los últimos 6 meses)**					
Sí	87	21,4	38,60 ± 7,62	t = 10,260	**0,000**
No	320	78,6	49,15 ± 11,15		
**Tiempo dedicado a las redes sociales (al día)**					
0–3 horas	259	63,6	45,49 ± 11,18	t = −3,340	**0,001**
4 horas o más	148	36,4	49,36 ± 11,26		
**Ejercicio (por semana)**					
Nunca^a^	90	22,1	37,95 ± 10,26	F = 67,181	**0,001** a<b<c
1–2 horas^b^	178	43,7	46,38 ± 8,10		
3 horas o más^c^	139	34,2	53,36 ± 11,50		
**Afiliación a un club o comunidad**					
Sí	116	28,5	52,40 ± 13,70	t = 5,545	**0,000**
No	291	71,5	44,71 ± 9,43		
**Elección voluntaria del departamento de obstetricia**					
Sí	323	79,4	49,27 ± 10,30	t = 9,049	**0,000**
No	84	20,6	37,78 ± 10,58		

**X:** Media, **DE:** Desviación estándar, **t**: Prueba t de Student, **F**: ANOVA unidireccional (post hoc: Tukey HSD), **GPA:** Promedio de notas.

La edad media de los participantes era de 20,64 ± 1,61 años. El promedio de notas era de 2,91 ± 0,47, el número medio de hermanos era de 2,26 ± 1,85, el número medio de cigarrillos fumados al día era de 8,47 ± 4,77, el número medio de vasos de alcohol consumidos a la semana era de 9,93±9,49, la puntuación media de la Escala de Autenticidad era de 46,90 ± 11,35, la puntuación media de la subescala de Autoalienación era de 14. 11 ± 4,41, la puntuación media de la subescala de Aceptación de la Influencia Externa fue de 19,46 ± 4,22, la puntuación media de la subescala de Vida Auténtica fue de 13,32 ± 5,63, la puntuación media de la Escala de Autocompasión fue de 2,79 ± 0,48, la puntuación media de la subescala de Autoafecto fue de 2,96 ± 0,81, la puntuación media de la subescala de Autojuicio fue de 2,43 ± 0. 92, subescala de Conciencia de Compartir puntuación media 2,92 ± 0,77, subescala de Aislamiento puntuación media 2,68 ± 0,95, subescala de Conciencia puntuación media 3,03 ± 0,83, subescala de Sobreidentificación puntuación media 2,77 ± 0,90, Inventario de Resolución de Problemas Interpersonales puntuación media 125,33 ± 21. 27, la puntuación media de la dimensión Enfoque negativo del problema fue 38,47 ± 11,05, la puntuación media de la dimensión Resolución constructiva de problemas fue 45,38 ± 8,45, la puntuación media de la dimensión Inseguridad fue 13,34 ± 4,84, la puntuación media de la dimensión Asumir responsabilidad fue 10,66 ± 3,32. La puntuación media de la subdimensión Enfoque Persistente fue de 17,47 ± 3,37 (Tabla 2).

**Tabla 2:** Distribución de variables continuas en el grupo de estudio – Balikesir, Ankara, Turquía, 2023.

Variables	X ± DE	Min–Max
**Edad**	20,64 ± 1,61	18–32
**GPA**	2,91 ± 0,47	1,00–3,90
**Número de hermanos**	2,26 ± 1,85	0–13
**Número de cigarrillos (al día)**	8,47 ± 4,77	1–20
**Cantidad de alcohol (por semana)**	9,93 ± 9,49	1–52
**Escala de autenticidad**	46,90 ± 11,35	12–81
Autoenajenación	14,11 ± 4,41	4–28
Aceptación de la influencia externa	19,46 ± 4,22	4–28
Vida auténtica	13,32 ± 5,63	4–28
**Escala de autocompasión**	2,79 ± 0,48	1–4,54
Autoafecto	2,96 ± 0,81	1–5
Autojuicio	2,43 ± 0,92	1–5
Conciencia de compartir	2,92 ± 0,77	1–5
Aislamiento	2,68 ± 0,95	1–5
Conciencia	3,03 ± 0,83	1–5
Sobreidentificación	2,77 ± 0,90	1–5
**Inventario de resolución de problemas interpersonales**	125,33 ± 21,27	50–200
Enfoque negativo del problema	38,47 ± 11,05	16–64
Resolución constructiva de problemas	45,38 ± 8,45	16–64
Inseguridad en uno mismo	13,34 ± 4,84	7–28
Falta de responsabilidad	10,66 ± 3,32	5–20
Enfoque insistente-persistente	17,47 ± 3,37	50–200

**X:** Media, **DE:** Desviación estándar, GPA: Promedio de calificaciones.

Cuando se analizó la relación entre las variables continuas y la Escala de Autenticidad en el grupo de investigación, se observó que no existían diferencias estadísticamente significativas entre las variables de GPA (r = 0,111, p = 0,026), puntuación de Autocompasión (r = 0,371, p = 0,000), puntuación de Autojuicio (r = 0,570, p = 0,000), puntuación de Aislamiento (r = 0,5022, p = 0,000), puntuación de Sobreidentificación (r = 0,492, p = 0000), Resolución de problemas interpersonales (r = 0,397, p = 0,000), Enfoque negativo de los problemas (r = 0,491, p = 0,000), Inseguridad (r = 0,296, p = 0,000), Falta de responsabilidad (r = 0,197, p = 0. 000), Insistencia-perdón (r = 0,296, p = 0,000), Insistencia-perdón (r = 0,296, p = 0,000),000), la puntuación de Enfoque Insistente-Persistente aumenta, la puntuación de Concienciación (r = −0,179, p = 0,000) y la puntuación de Autocompasión disminuye (r = −0,207, p = 0,000), la puntuación de la Escala de Autenticidad aumenta de forma estadísticamente significativa. No hay diferencias significativas según otras variables y dimensiones (p > 0,05) (Tabla 3).

**Tabla 3:** Relación entre variables continuas y nivel de autenticidad – Balikesir, Ankara, Turquía, 2023.

Variables	r	p
**Edad**	−0,003	0,948
**GPA**	0,111	**0,026**
**Número de hermanos**	−0,056	0,260
**Número de cigarrillos**	−0,179	0,209
**Cantidad de alcohol**	−0,208	0,175
**Escala de autocompasión**	0,371	**0,000**
Autoafecto	−0,207	**0,000**
Autojuicio	0,570	**0,000**
Conciencia de compartir	−0,077	0,121
Aislamiento	0,522	**0,000**
Conciencia	−0,179	**0,000**
Sobreidentificación	0,492	**0,000**
**Inventario de resolución de problemas interpersonales**	0,397	**0,000**
Enfoque negativo del problema	0,491	**0,000**
Resolución constructiva de problemas	0,056	0,261
Inseguridad en uno mismo	0,296	**0,000**
Falta de responsabilidad	0,197	**0,000**
Enfoque insistente-persistente	0,132	**0,008**

**X:** Media, **DE:** Desviación típica, **r:** Coeficiente de correlación de Pearson, **GPA:** Promedio de notas.

Las variables que resultaron estadísticamente significativas por los análisis univariantes y la relación entre las variables independientes significativas en la bibliografía y la variable dependiente se evaluaron mediante un análisis de regresión lineal utilizando el método Enter. Las variables incluidas en el modelo explican el 52% del cambio en la Escala de Autenticidad (R^2^ = 0,538, R^2^ajustada = 0,524, F = 38,206 p = 0,000).

Como resultado del análisis de regresión lineal, se observó que la puntuación de la Escala de Autenticidad era significativamente más baja en los alumnos que estudiaban en una universidad de fundación (β = 2,671; IC del 95%: 0,96; 4,37), en aquellos cuyos padres estaban separados/muertos (β = −5,083; IC del 95%: −7,99; −2,17), en aquellos cuyas madres tenían estudios primarios o menos (β = −4,478; IC del 95%: −6,28; −2,67), en aquellos que experimentaron algún tipo de violencia en los últimos 6 meses (β = −5. 272; IC 95% −7,23; −3,30), aquellos que pasan menos tiempo en las redes sociales (β = 1,006; IC 95% 0,46; 1,54), aquellos cuya duración del ejercicio disminuye (β = −2,648; IC 95% −3,43; −1. 86), las que no eran miembros de un club/comunidad (β = −3,236; IC 95% −5,00; −1,46), y las que no eligieron el programa de matronas voluntariamente (β = −5,383; IC 95% −7,47; −3,29). Además, a medida que disminuye la puntuación de Autocompasión (β = 0,102; IC 95% 0,02; 0,17) y disminuye la puntuación de Resolución de problemas interpersonales (β = 0,081; IC 95% 0,03−0,12), también disminuye la puntuación de Autenticidad de las participantes (Tabla 4).

**Tabla 4:** Evaluación de las variables independientes y puntuación de autenticidad según el análisis de regresión lineal – Balikesir, Ankara, Turquía, 2023.

Variables	ß	S.E.	Estándar ß	p	%95 C.I.	
					Baja	Superior
**Universidad**	2,671	0,867	0,110	**0,002**	0,96	4,37
**GPA**	0,957	0,863	0,039	0,268	–0,73	2,65
**Estructura familiar**	–5,083	1,482	–0,121	**0,001**	–7,99	–2,17
**Nivel de estudios de la madre**	–4,478	0,918	–0,195	**0,000**	–6,28	–2,67
**Nivel de estudios del padre**	1,505	0,913	0,064	0,100	–0,29	3,29
**Ha sufrido algún tipo de violencia en los últimos 6 meses**	–5,272	1,000	–0,191	**0,000**	–7,23	–3,30
**Tiempo dedicado a las redes sociales (al día)**	1,006	0,276	0,127	**0,000**	0,46	1,54
**Ejercicio (por semana)**	–2,648	0,399	–0,258	**0,000**	–3,43	–1,86
**Afiliación a un club o comunidad**	–3,236	0,900	–0,129	**0,000**	–5,00	–1,46
**Elección voluntaria del departamento de obstetricia**	–5,383	1,063	–0,192	**0,000**	–7,47	–3,29
**Escala de autocompasión**	0,102	0,039	0,114	**0,010**	0,02	0,17
**Inventario de resolución de problemas interpersonales**	0,081	0,024	0,152	**0,001**	0,03	0,12

ß: Coeficiente de regresión, **E.S.**: Error estándar, **p**: Valor de significación, IC: Intervalo de confianza, **GPA:** Promedio de notas.

R2ajustada = 0,524, F= 38,206, p = 0,000.

## DISCUSIÓN

Los individuos auténticos son creativos, están abiertos a la innovación y confían en sus experiencias e interioridades^(18)^. La capacidad de las parteras para ser individuos auténticos afecta significativamente a la calidad de los cuidados, la cooperación interprofesional, el compromiso organizativo y la satisfacción laboral^(6,19,20)^. En este estudio, que se llevó a cabo para determinar la relación entre la autocompasión, las habilidades interpersonales de resolución de problemas y los niveles de autenticidad en estudiantes de partería, se determinó que existía una relación positiva significativa entre la autocompasión y las habilidades de resolución de problemas y los niveles de autenticidad de las estudiantes de partería. En nuestro estudio, el nivel de autenticidad de las estudiantes de partería resultó ser medio, coincidiendo con la bibliografía. El hecho de que la autenticidad no sea alta puede deberse a las escasas oportunidades de realizar prácticas debido al creciente número de estudiantes, a la incapacidad de los estudiantes de partería de pasar un tiempo limitado con mujeres en el ámbito de la práctica en Turquía y al estatus de la partería. En el estudio realizado por Bloxsome y Bayes, se destaca la importancia de permanecer con mujeres para sentir la identidad innata, la identidad que surge del entorno y la autenticidad^(21)^.

Además, las puntuaciones de autenticidad aumentan con el promedio académico. Este resultado sugiere que el éxito de las estudiantes en los estudios de partería también mejora su autenticidad. No hay ningún estudio en la literatura que examine el nivel de autenticidad de las estudiantes de partería. En cambio, hay estudios que examinan el nivel de autenticidad de los estudiantes universitarios. Estos estudios se realizaron principalmente con estudiantes del campo de las ciencias sociales. Los resultados de estos estudios son similares a los nuestros, y se determinó que la puntuación de autenticidad de los estudiantes universitarios se situaba en un nivel medio^(1,2,3,22)^. Cuando se examinan los estudios realizados con diferentes métodos en la bibliografía, se observa que la autenticidad aumenta a medida que aumentan las relaciones de los estudiantes de partería con los proveedores de servicios sanitarios^(23)^ y a medida que aumentan las oportunidades de aplicación, como la simulación^(24)^. En este sentido, se debería intentar aumentar la autenticidad de los estudiantes de partería.

Nuestro estudio descubrió que la edad, el curso, el número de hermanos, la percepción de ingresos, el lugar de residencia, la estructura familiar, el hábito de fumar y el consumo de alcohol no afectaban a la autenticidad. Aunque no hay estudios sobre estudiantes de partería en la bibliografía, se ha visto que la edad de las estudiantes de enfermería y de las enfermeras afecta al nivel de autenticidad^(1,25)^. La educación en partería requiere un alto nivel de conocimientos y habilidades profesionales debido a la naturaleza de la profesión; por lo tanto, es una de las profesiones en las que se experimenta con frecuencia ansiedad por estar en un entorno educativo diferente y agotamiento emocional^(26)^. Medir la autocompasión de las estudiantes durante su formación, determinar los factores que la afectan e incrementarla aumentará el desarrollo profesional de las estudiantes de partería. En la literatura, se informa de que cuanto mayor es el nivel de autenticidad, mayor es la autocompasión de los individuos^(18,27)^. En nuestro estudio, a medida que aumenta el nivel de autocompasión, también aumenta el nivel de autenticidad. Desde este punto de vista, puede decirse que ser un buen amigo de uno mismo aumenta la capacidad de actuar de acuerdo con los propios sentimientos y pensamientos con la mayor independencia posible de los demás, revelando el verdadero yo. En un estudio realizado con estudiantes de enfermería en la literatura similar a este estudio, se encontró que la autenticidad aumentaba a medida que aumentaba la autocompasión^(1)^. En nuestro estudio se encontró un resultado similar, y a medida que aumentaba la autocompasión de las estudiantes de partería, también lo hacía su nivel de autenticidad. Como resultado de un estudio realizado en estudiantes universitarias, se determinó que las subdimensiones de autenticidad explicaban el 28% del nivel de autocompasión^(18)^. Añadir asignaturas que mejoren la autocompasión a los programas educativos de los estudiantes de partería puede prepararlos para gestionar los factores de estrés, promover el autocuidado y mejorar la atención compasiva prestada a los demás.

La prestación de cuidados de partería individualizados requiere habilidades de pensamiento crítico y analítico, creatividad y la capacidad de producir soluciones prácticas a los problemas a los que se enfrentan. En este contexto, las estudiantes de partería necesitan experiencias de aprendizaje en las que se adopte un enfoque de resolución de problemas para desarrollar sus conocimientos, habilidades y actitudes. En nuestro estudio, se observó que las estudiantes de partería tenían una capacidad moderada para resolver problemas. Cuando se examinaron las habilidades de resolución de problemas de las estudiantes de partería en la bibliografía, se determinó que sus habilidades de resolución de problemas se encontraban en un nivel medio^(12,14,28)^. Sin embargo, ningún estudio bibliográfico examina el efecto de las habilidades de resolución de problemas en la autenticidad. En nuestro estudio, se observó que el nivel de autenticidad aumentaba a medida que aumentaban las habilidades de resolución de problemas. Esta situación se interpreta como que los estudiantes planifican cuidados de partería creativos e innovadores a medida que mejoran sus habilidades de resolución de problemas. En este contexto, se recomienda utilizar múltiples métodos de enseñanza, como el aprendizaje basado en la simulación, el aprendizaje aplicado, el uso reflexivo y el aprendizaje basado en proyectos, que aumentan las habilidades de resolución de problemas, para incrementar la resolución de problemas, la autodirección y la autenticidad de las estudiantes de partería e integrarlos en la enseñanza universitaria.

Para evaluar correctamente este estudio, hay que tener en cuenta algunas limitaciones. En primer lugar, una limitación del estudio es que los resultados se basan en datos autoinformados (cuestionarios autocumplimentados). En segundo lugar, este estudio sólo incluyó un número limitado de estudiantes de partería de dos universidades diferentes, por lo que los autores reconocen que es poco probable que la muestra sea representativa de la población. En futuras investigaciones, se recomienda realizar estudios similares con muestras más amplias y diversas.

## CONCLUSIÓN

Los resultados revelaron que los participantes tenían niveles moderados de autenticidad, mientras que su autocompasión y sus habilidades para resolver problemas eran bajas. Se descubrió que el aumento de la autocompasión y de la resolución de problemas interpersonales influía positivamente en la autenticidad. Los factores que afectaron a la autenticidad fueron: la universidad en la que se formó el participante; la estructura familiar; el nivel educativo materno; la exposición a algún tipo de violencia en los últimos 6 meses; el tiempo dedicado a las redes sociales; la pertenencia a un club, y la elección voluntaria de la profesión. A medida que aumentan las habilidades de resolución de problemas interpersonales y la autocompasión, también aumenta la autenticidad.

Se recomienda garantizar la integridad familiar, aumentar la educación de los padres, incrementar los niveles de actividad física de los estudiantes, fomentar la participación en clubes/comunidades y aumentar la capacidad de resolución de problemas y la sensibilidad.

Se recomienda que los programas educativos integren múltiples métodos de enseñanza como el aprendizaje basado en simulaciones, aplicado, reflexivo y basado en proyectos- para mejorar la autenticidad, la autocompasión y las habilidades de resolución de problemas de los estudiantes. También puede ser eficaz incluir intervenciones dirigidas a la autocompasión y la resolución de problemas en el asesoramiento individual/grupal y en las prácticas psicoeducativas. Se recomiendan futuros estudios experimentales para evaluar el impacto de dichos programas sobre la autenticidad, y la investigación debería ampliarse a diferentes grupos de edad y niveles educativos.

## Data Availability

Los datos relacionados con este estudio están disponibles a través del autor correspondiente previa solicitud justificada.
